# Preserved repetition in thalamic afasia. A pathophysiological hypothesis

**DOI:** 10.1590/1980-57642018dn13-020015

**Published:** 2019

**Authors:** Ricardo Nitrini, Leandro T. Lucato, Maria C. Sitta, Maíra O. Oliveira, Daniel Ciampi de Andrade, Valquiria A. Silva, Camila G. Carneiro, Carlos A. Buchpiguel

**Affiliations:** 1 Department of Neurology, University of São Paulo Medical School, São Paulo, SP, Brazil.; 2 Department of Radiology, University of São Paulo Medical School, São Paulo, SP, Brazil.; 3 Geriatrics Section of the Department of Internal Medicine, University of São Paulo Medical School, São Paulo, SP, Brazil.

**Keywords:** aphasia, thalamic aphasia, repetition, neuroimaging, subcortical aphasia, afasia, afasia talâmica, afasia subcortical, repetição neuroimagem

## Abstract

The ability to repeat words is almost always preserved in thalamic aphasia. The pathophysiology of both thalamic aphasia and preservation of repetition are not fully understood. In a case of severe aphasia with preserved repetition after a left thalamic hemorrhage, MRI disclosed left thalamic lesion and loss of fractional anisotropy in the left centrum semiovale. FDG-PET showed severe hypometabolism in the left cerebral hemisphere, except for superior and transverse temporal gyri, calcarine fissure and frontopolar regions. Primary sensory function may be less functionally dependent on thalamic connections than heteromodal and paralimbic areas, which have connections with several thalamic nuclei. The extensive cortical hypometabolism due to diaschisis may have been responsible for the severity of the aphasia, whereas the less severe reduction of metabolism in the superior and transverse temporal gyri, and also, albeit less evident, in Broca’s area, might explain the preservation of repetition.

## RESUMO.

A capacidade de repetir palavras é quase sempre preservada na afasia talâmica. A fisiopatologia da afasia talâmica assim como a da preservação da repetição não são totalmente compreendidas. Em um caso de afasia grave com repetição preservada após hemorragia talâmica esquerda, a RM revelou lesão talâmica esquerda e perda de anisotropia fracionada no centro semioval. O FDG-PET revelou hipometabolismo grave no hemisfério cerebral esquerdo, exceto nos giros temporais superiores e transversos, fissura calcarina e regiões frontopares. A função sensorial primária pode ser menos funcionalmente dependente das conexões talâmicas do que as áreas heteromodais e paralímbicas, que têm conexões com vários núcleos talâmicos. O hipometabolismo cortical extenso devido à diasquise pode ter sido responsável pela gravidade da afasia, enquanto a redução menos severa do metabolismo nos giros temporal superior e transverso, e também, embora menos evidente, na área de Broca, poderia explicar a preservação da repetição.

Cognitive functions, particularly language, have always been considered “higher cortical functions”. In this context, aphasia due to thalamic lesions, although described over one hundred years ago,^1^^,^ 2 were considered inconsistent or dubious until the 1970’s. For instance, no mention of aphasia due to thalamic lesions can be found in two of the most influential European books on cognitive neurology of the 1960’s.^3^^,^ 4

The advent of CT-scans in the 1970’s allowed easier topographic diagnosis of thalamic lesions. Several reports of thalamic aphasia have since been published.^5^^-^ 7 It was usually described as an atypical aphasia,^8^^-^ 10 with the remarkable finding of preservation of word repetition.^8^^,^ 10 ^,^ 11 ^,^ 12

Gaps in our understanding of thalamic aphasia remain. The thalamus is an important hub of cortico-striato-thalamic-cortical loops,^8^^,^ 10 ^,^ 12 which may explain the aphasia after thalamic lesions. Also, thalamic nuclei are part of the ascending reticular activating system^13^^,^ 14 In thalamic lesions, the activity of the neurons of the cerebral cortex is decreased due to reduction of afferent excitability, a phenomenon called diaschisis.This phenomenon explains changes in both functional and structural properties in areas that are distal to, but previously connected with, the lesion site.^15^^,^ 16

Furthermore, the reason for preservation of repetition of words often seen in thalamic aphasia is also unclear.

We studied a case of thalamic hemorrhage and, based on analyses of the clinical features and neuroimaging findings, we propose a hypothesis for the preservation of repetition.

## CASE REPORT

**Figure 1:**
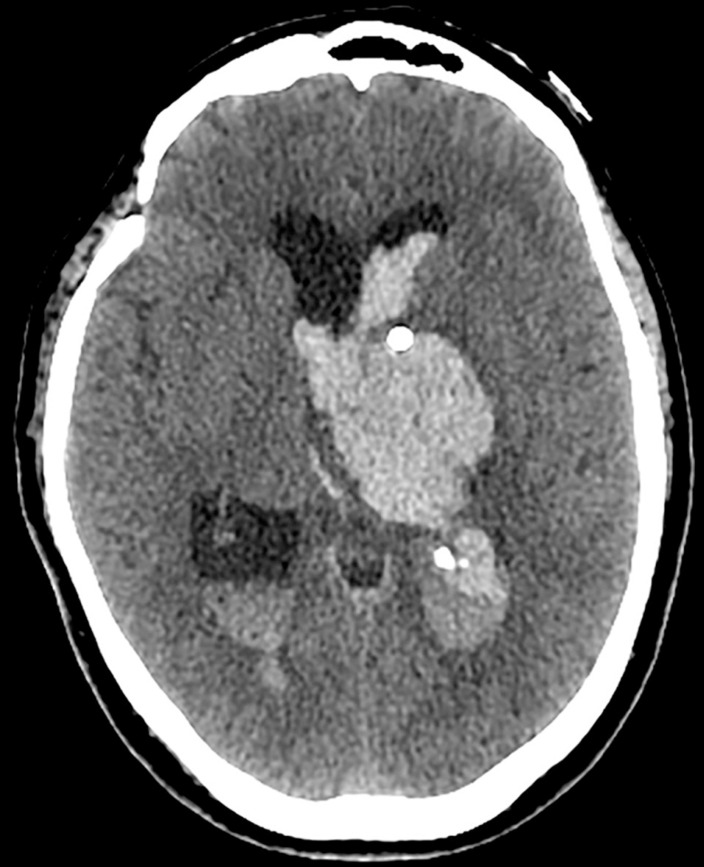
Head CT reveals an intracerebral hemorrhage of approximately 50 milliliters with its center in the left thalamus, which ruptured into the ventricles. There is dilation of the lateral ventricles and a small midline shift. Also note an intraventricular catheter for external ventricular drainage.

A 59-year-old, right-handed woman, who had been treated for moderate arterial hypertension, had an acute episode of right-side hemiplegia. A brain CT revealed an intracerebral hemorrhage of approximately 50 milliliters with its center in the left thalamus, which had ruptured into the ventricles (Figure 1). She was submitted to external ventricular drainage with continuous monitoring of intracranial pressure. The patient remained unconscious for several weeks and was hospitalized for almost five months.

She was seen at the outpatient clinic 19 months after the stroke. She had remained at home since leaving hospital, where she experienced severe limitation in daily activities and was still using diapers. She was always in a good mood, seemingly unaware of her condition.

At examination, she was in a wheelchair, with neglect of the right visual field, right-sided hemiplegia and hemianesthesia. She was unable to perform on command or to imitate simple gestures with her left arm.

Her spontaneous speech was very poor, restricted to simple words or monosyllables. When she tried to say something else there were many phonemic paraphasias and neologisms. Palilalia was also frequent. She was able to understand and respond to simple commands such as “open your mouth”, but perseveration ensued almost immediately. Naming was also severely impaired. She was able to name only one out of ten simple drawings and she also had severe difficulty singling out an object after hearing its name. Her performance fluctuated on almost all tasks, although was consistently very poor.

Repetition of single words or familiar short sequence of words (such as the name of the street where she had been living for years) was preserved.

She was able to repeat 4 digits forwards, but none backwards. Semantic verbal fluency was zero and she scored 3 on the phonemic (FAS) test. She was able to read simple phrases aloud such as “close your eyes”, but she did not obey the command. She was unable to write, not even single letters, or to copy single figures, when closing-in phenomena was observed.

Her speech improved for a few seconds when she was asked about her only son, but after only two or three short phrases, her speech again became unintelligible and non-fluent. Besides her ability to repeat words, she also could sing old songs together with her caregivers.

### Neuroimaging

**Figure 2:**
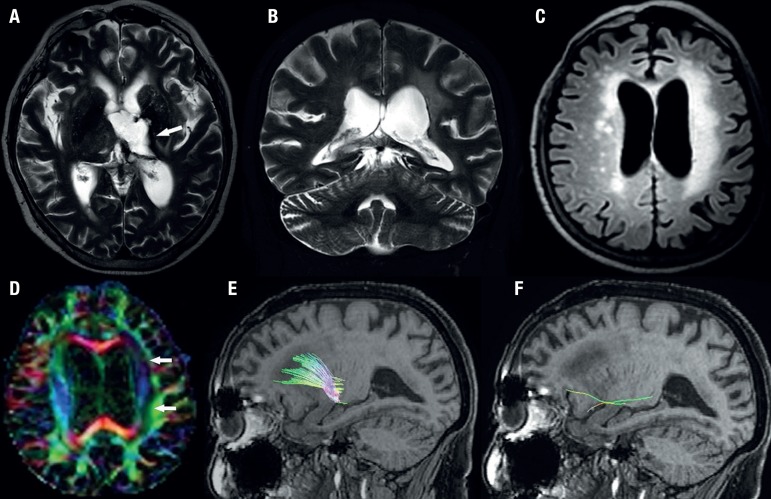
Axial T2-weighted image (A) shows a residual cavity related to previous intraparenchymal hemorrhage centered in the left thalamus (arrow). Coronal T2-weighted image (B) discloses slight atrophy of the right cerebellar hemisphere. Axial FLAIR image (C) demonstrates a smaller left cerebral hemisphere compared to the contralateral side; also note the confluent white matter hyperintensity in the left centrum semiovale. Color fractional anisotropy map (D) clearly shows loss of anisotropy in the left centrum semiovale, characterized by a decrease in signal compared to the contralateral white matter. Tractography (E and F) focusing on anterior thalamic radiations demonstrates normal appearance on the right (E), while there is almost no clear fiber identification on the left (F).

MRI showed a residual cavity in the left thalamus with a confluent white matter hyperintensity in the left centrum semiovale. The left hemisphere was slightly smaller than the right hemisphere. Color fractional anisotropy map revealed loss of anisotropy in the left centrum semiovale, and tractography focusing on anterior thalamic radiations showed normal appearance on the right side, yet almost no clear fiber identification on the left side. (Figure 2A-F).

The 18F-Fluor-dexoxi-glucose PET (18F-FDG-PET) scan showed severe glycolytic hypometabolism in the left cerebral hemisphere and contralateral cerebellar hemisphere. Coronal views showed that metabolism was less reduced in the transverse cortical gyri. On the lateral surface of the left hemisphere, only the occipital cortex, frontopolar region and a small area corresponding to the superior temporal gyrus were less involved when compared with the database control. A Z-score mapping system showed a marked reduction of meta bolism in the left cortical hemisphere, but an area corresponding to the left superior temporal gyrus and a small area of the left inferior frontal gyrus had better preserved metabolism than other areas in the left hemisphere. Analyses of the images using Statistical Parametric Mapping (SPM^8^) showed better preserved metabolism in the superior temporal gyrus, in a region of the inferior frontal gyrus roughly corresponding to Broca’s area, compared to a normal volunteer database group, (Figures 3A-C) and also in the region corresponding to the calcarine fissure (Figure 4 - shown at: http://www.demneuropsy.com.br/imagens/v13n2-fig04.jpg). She did not improve with transdermal rivastigmine and with repetitive transcranial magnetic stimulation (r-TMS), which was performed with a MagPROX100 device (Magventure® Tonika Elektronik, Farum, Denmark) at 10Hz, using a butterfly double-cone D-B80 cooled coil, held tangentially to the scalp using previously described protocols.^17^ The total treatment consisted of sessions five days per week for two weeks. Neuropsychological evaluations were performed before, after the first and after the second weeks. 18F-FDG-PET scans performed before and immediately after the end of the r-TMS also failed to show a difference.

**Figure 3A:**
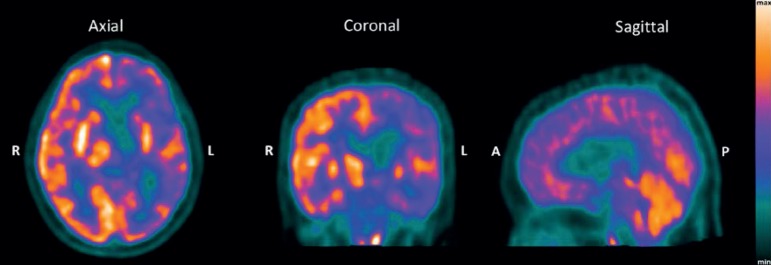
Positron emission tomography (PET) with ^18^F-FDG. Note the marked reduction in glucose uptake in the left cerebral cortex, including the left thalamus and striatum. The area corresponding to the transverse temporal gyri has more preserved metabolism, evident in the axial and coronal planes. R: right; L: left; A: anterior; P: posterior.

**Figure 3B:**
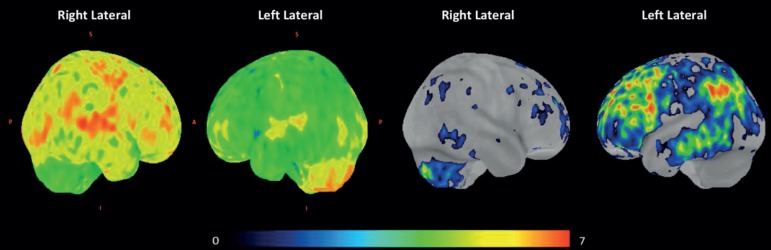
^18^F-FDG PET data (sagittal plane) were analyzed with Z-score mapping system, using software Cortex ID, GE Healthcare. On the left, note the 3D images showing lower metabolism in the left cerebral cortex compared to the right cerebral cortex. An area corresponding to the left superior temporal gyrus and a small area of the left inferior frontal gyrus have more preserved metabolism than other areas in the left hemisphere. In fourth image on the right, note colored areas highlighting the most significant regions with cortical metabolic deficits in the left hemisphere. A: anterior; P: posterior; S: superior; I: inferior.

**Figure 3C:**
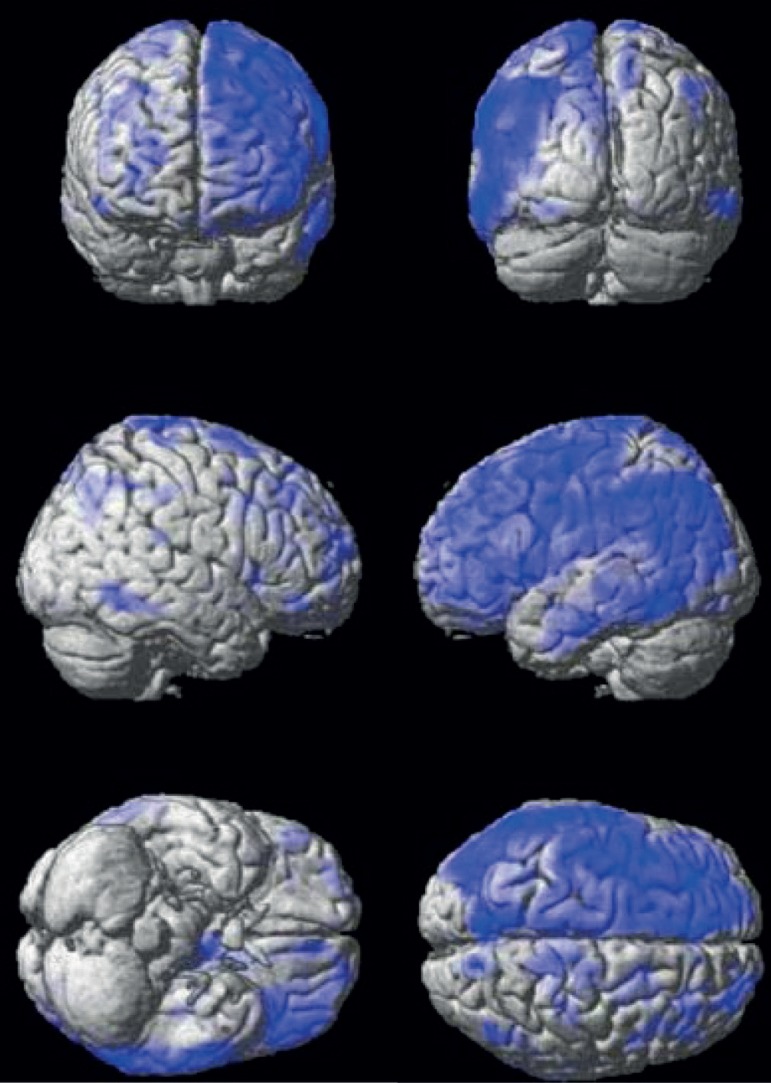
^18^F-FDG PET 3D images (in order from top to bottom: Coronal, Sagittal and Axial planes) were analyzed with Statistical Parametric Mapping (SPM), threshold p < 0.01, uncorrected for multiple comparisons. Blue areas are the illustrative anatomical location of voxels with statistically significant regional brain glucose metabolism reduction compared to a normal volunteer database group. Note the more preserved metabolism in the left superior temporal gyrus and in a region of the left inferior frontal gyrus compared to other areas of the left cortex.

## DISCUSSION

This patient presented with right-sided hemiplegia, probably due to involvement of the internal capsule, and with other signs that suggested involvement of the left cerebral cortex, including severe aphasia, left-sided apraxia and right visual field neglect. PET imaging showed that the metabolism was severely impaired in almost the entire left brain hemisphere, except for a few regions, which included the calcarine region and the superior and transverse temporal gyri. These findings suggest that the main visual and auditory pathways might have remained undamaged.

### Aphasia: clinical features

This patient had an atypical aphasia with characteristics suggesting mixed transcortical aphasia, which involves both sensory and motor components. Poor comprehension with jargon aphasia is described in sensory transcortical aphasia, whereas low spontaneous speech is a characteristic of motor transcortical aphasia.^18^ According to the classical view, mixed transcortical aphasia is caused by anoxic, toxic or ischemic lesions in the left cortical areas located posteriorly to Heschl’s gyri and anteriorly to Broca’s area, sparing the perisylvian language areas, explaining the preservation of the ability to repeat words.^18^ This classical view has been questioned following observations of mixed transcortical aphasia with extensive lesions to the left hemisphere, where the perisylvian language areas were not spared.^19^^,^ 20 In these cases, there are data suggesting that the contralateral hemisphere may mediate repetition abilities.^20^

In the case described here, language was not restricted to repetition, but all other language features were severely impaired, suggesting an extensive cortical lesion. Some authors have described transcortical features in thalamic aphasia,^6^^,^ 11 while others regard thalamic aphasia as an atypical aphasia^7^^-^ 10

### Aphasia: pathophysiology

Many hypotheses have been put forward to explain the occurrence of aphasia in thalamic lesions (see review by Crosson, 2013).^10^ Involvement of the cortico-striato-pallidum-thalamus-cortical loops^8^^,^ 21 and diaschisis due to the reduction of impulses from the thalamus to cortical networks^15^ are the most frequently postulated theories.

Studies using SPECT (single photon computed tomography)^22^^,^ 23 and with PET have revealed cortical hypoperfusion in the thalamic aphasia, suggesting that diaschisis could be the main causative factor of aphasia.^15^^,^ 24

In the present case, 18F-FDG-PET showed diffuse severe cortical hypometabolism in almost all left hemisphere cortical areas, with relative preservation of metabolism in perisylvian language areas (Figure 3A-C). Diaschisis may be responsible for the clinical features of the aphasia in this case.

### Preservation of repetition

Our hypothesis is that the ability to repeat words seen in this case is due to the relatively preserved metabolism in the perisylvian language areas. Primary sensory and motor areas have the most restricted connections with thalamic nuclei (and with medial and lateral geniculate nuclei for auditory and visual pathways, respectively), whereas the heteromodal and paralimbic areas have heterogeneous connections with several thalamic nuclei, including those that are part of the reticular activating system.^14^^,^ 25 ^,^ 26 In this sense, primary areas are less dependent on thalamic impulses.

To conclude, thalamic aphasia may be predominantly due to diaschisis, as proposed by other studies. The preser vation of the ability to repeat words after thalamic lesions might be explained by the relative preservation of brain metabolism in the perisylvian language areas, given these areas tend to have less thalamic connectivity (and thus less dependent on thalamic impulses) than heteromodal and paralimbic areas. Clearly no hard conclusion can be reached from the study of a simple case, but ideas raised by a case study can be subsequently tested in case series or with more advanced tools.
